# From biomarker to clinical utility: translating the advanced lung cancer inflammation index into a machine learning-driven risk stratification tool for colorectal cancer

**DOI:** 10.1186/s12967-025-07494-z

**Published:** 2025-11-26

**Authors:** Ming Gao, Ying Li, Huimei Wang, Jinming Zhang, Guangxun Zhang, Nan Zhang

**Affiliations:** Department of Gastroenterology, The First Hospital of Jilin University, No.1 Xinmin Street, Changchun, 130012 China

**Keywords:** Advanced lung cancer inflammation index, Colorectal cancer, National health and nutrition examination survey, Boruta algorithm, Machine learning algorithm

## Abstract

**Background:**

Both nutrition and inflammation have been implicated in the pathogenesis of colorectal cancer (CRC), but most previous studies have examined these factors separately. This study aimed to explore the combined association of inflammation and nutritional status with CRC.

**Methods:**

This study selected 101,316 subjects from the National Health and Nutrition Survey (NHANES) conducted from 1999 to 2018. First, weighted logistic regression was used to measure the association between the advanced lung cancer inflammation index (ALI) and CRC. Then, restricted cubic splines (RCS) were used to capture the dose-response curve, and the predictive power of the model was calibrated by the ROC curve. Subsequently, robustness was verified through subgroup and interaction analyses. Furthermore, random forest analysis combined with the Boruta algorithm was employed to identify CRC-related factors. Subsequently, a machine learning(ML) prediction framework is constructed, and the black box of the optimal model is disassembled using SHAP values to endow it with interpretability.

**Results:**

In the fully adjusted model, each unit increase in log-transformed ALI was associated with a 20.9% reduction in CRC risk (OR = 0.791; 95% CI: 0.628–0.997; *p* = 0.047). Participants in the highest log-ALI quartile had a 46.2% lower risk compared to those in the lowest quartile (OR = 0.538; 95% CI: 0.344–0.842; *P* = 0.007). The fully adjusted model demonstrated strong discriminative ability (AUC = 0.848). RCS analysis confirmed a linear dose-response relationship (P for nonlinearity = 0.731). The robustness of these findings was supported by subgroup and sensitivity analyses. Random forest analysis coupled with the Boruta algorithm identified log-ALI as a strong predictor. Among seven machine learning models evaluated, the LightGBM algorithm achieved the highest and most stable predictive performance (AUC = 0.870). SHAP analysis confirmed log-ALI as the most important protective feature.

**Conclusion:**

This study demonstrates that higher ALI levels, indicative of better nutritional and inflammatory status, are significantly associated with a lower risk of CRC. The optimized ML model based on ALI shows promise as a cost-effective tool for CRC risk stratification.

**Supplementary Information:**

The online version contains supplementary material available at 10.1186/s12967-025-07494-z.

## Introduction

Colorectal cancer (CRC) poses a significant global public health challenge. It is the third most commonly diagnosed malignancy and the second leading cause of cancer-related mortality worldwide [1]. Recent data from 2024 indicate approximately 1.8 million new cases and 881,000 deaths globally [2]. If this curve is followed at full speed, the number of cases will have to increase by another 63% by 2040. Furthermore, the increasing incidence of early-onset CRC, combined with rising healthcare costs, imposes a substantial economic burden on healthcare systems worldwide.

A concerning trend of increasing early-onset CRC, coupled with rising incidence projections, places a substantial economic and clinical burden on healthcare systems [3]. In recent years, the global rise in obesity has emerged as a critical risk factor for CRC [4]. Excess adiposity contributes to CRC initiation and progression through mechanisms involving chronic low-grade inflammation, insulin signaling dysregulation, and redox imbalance [5]. Obesity also disrupts hormonal homeostasis, further amplifying adipose tissue–mediated inflammatory pathways [6]. Moreover, inadequate nutrition and persistent inflammation play pivotal roles in the pathogenesis of gastrointestinal malignancies. Inflammatory processes are recognized as key determinants of tumor initiation, progression, and prognosis [7]. Chronic inflammation may impair immune surveillance and promote a tumor-supportive microenvironment [8]. Emerging evidence has identified several inflammation-related markers as reliable prognostic indicators for CRC, such as the neutrophil-to-albumin ratio (NPAR) [9], which shows a strong association with clinical outcomes. Moreover, poor nutritional status exacerbates persistent intestinal inflammation, thereby promoting malignant transformation [10]. Concurrently, reduced serum albumin levels are closely associated with cancer-related cachexia, characterized by progressive weight loss and muscle wasting [11]. Therefore, a comprehensive assessment of the interplay between inflammation and nutrition may provide valuable insights for clinical strategies aimed at reducing CRC risk.

Clinically, the management of CRC continues to pose significant challenges. Current therapeutic strategies mainly adopt a multimodal approach centered around surgical resection [12]. Although chemotherapy is widely utilized for its practicality [13], its efficacy is often limited by inadequate drug accumulation and multidrug resistance [14, 15]. The development of anti-angiogenic agents such as thalidomide marked a therapeutic advance [16, 17], and more recent combinations with immune checkpoint inhibitors have shown synergistic efficacy in advanced CRC [18]. Concurrently, nanomedicines have emerged as promising platforms for their tumor-targeting and controlled-release capabilities, demonstrating selective anti-CRC activity and potential to overcome resistance when combined with conventional drugs [19–21]. Against this backdrop, the identification of biomarkers capable of accurately predicting treatment response and prognosis is essential to guide personalized therapeutic strategies [22].

The systemic inflammatory response acts as a critical mediator linking various metabolic abnormalities and intricately interacts with pathological conditions such as obesity and insulin resistance [23]. To better capture this complex interplay, the Advanced Lung Cancer Inflammation Index (ALI) was developed as an innovative composite scoring system. Originally proposed by Jafri et al. for prognostic assessment in non-small cell lung cancer [24], ALI integrates inflammatory markers with nutritional status indicators, thereby overcoming the limitations of conventional single-parameter biomarkers [17]. Subsequently, the predictive utility of ALI has been validated across a spectrum of malignancies, including breast, hepatocellular, and gastric cancers [25–27]. More recently, its application has expanded to non-oncological chronic diseases such as hypertension, heart failure, coronary artery disease, and Crohn’s disease [28–31]. The unique clinical value of ALI derives from its integration of key parameters: it combines nutritional metrics such as body mass index and serum albumin with the neutrophil-to-lymphocyte ratio (NLR) [32], and has established prognostic value in metabolic diseases like diabetes [33]. This synthesis of immunological and nutritional dimensions provides a more comprehensive assessment of disease pathophysiology [31]. By integrating nutritional and inflammatory pathways through readily available parameters such as albumin, NLR, and BMI, ALI offers a clinically accessible approach aligned with the Bench-to-Bedside (B2B) paradigm in translational medicine.

However, evidence on the predictive value of ALI specifically in CRC remains limited. To address this knowledge gap, we utilized the nationally representative NHANES cohort to comprehensively investigate the association between ALI and CRC risk. Our approach was to first lay a solid foundation with traditional statistics and then leverage machine learning (ML) to build visual models, representing a novel approach with potential to enhance clinical diagnosis and treatment strategies. These findings position ALI as a promising biomarker for CRC risk assessment. However, further validation in larger, prospective cohorts is warranted to confirm its clinical utility.

## Materials and methods

### Study participants and research design

The NHANES employs a sophisticated stratified sampling design, administered by the Centers for Disease Control and Prevention (CDC), to assess population health in the United States. This nationally representative study has collected comprehensive data on physiological parameters and dietary patterns from participants of all ages through biennial surveys since 1999. Each cycle systematically records sociodemographic information, dietary intake, clinical examination results, biochemical measurements, and questionnaire responses. Ethical approval for all study procedures was granted by the Research Ethics Review Board of the National Center for Health Statistics (NCHS), and written informed consent was obtained from all participants prior to enrollment.

**Fig. 1:**
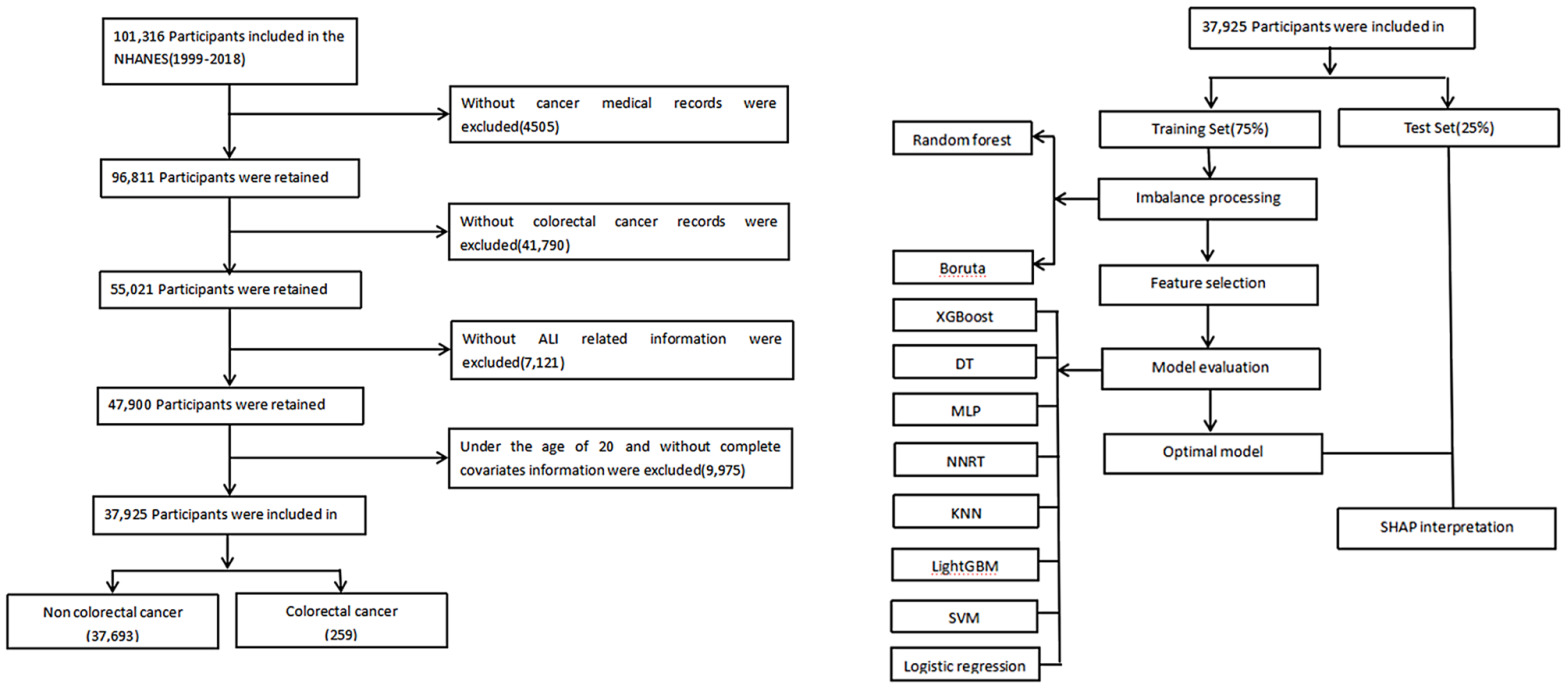
Flowchart of eligible study participants

Our study utilized NHANES datasets from 1999 to 2018, applying rigorous participant selection based on predefined exclusion criteria. Individuals were excluded if they: (1) were under 20 years of age; (2) lacked confirmed malignancy status; (3) had incomplete biomarker data required for ALI calculation (neutrophil count, lymphocyte count, serum albumin, or BMI); or (4) had missing values for any covariates, including demographic, lifestyle, socioeconomic, or comorbidity variables. Following these exclusions, the final analytical cohort included 37,952 participants who met all study criteria (Fig. 1).

### Diagnostic criteria for CRC

The history of tumors was provided by the Medical Condition Section (MCQ) in the NHANES questionnaire. First, screen through the MCQ220 with the question “Did the doctor ever tell you that you have A tumor or a malignant disease?” Then, use the MCQ230 A-D to follow up with the question “What type of cancer is it exactly?” These two steps help to determine the malignant status. Participants were classified as CRC cases only if they reported a malignancy and specified colon or rectal cancer. The control group included individuals with other cancer types, no history of cancer, or a combination of CRC and other malignancies. Data were collected via in-person interviews or computer-assisted systems, with trained personnel applying strict quality control procedures to ensure accuracy and minimize errors.

### Assessment of ALI

The ALI was calculated as serum albumin (g/dL) × BMI (kg/m²) / NLR [34]. The NLR was derived from the complete blood count by dividing the absolute neutrophil count by the absolute lymphocyte count. BMI was computed as weight in kilograms divided by the square of height in meters. All laboratory procedures adhered to the standardized protocols outlined in the NHANES Laboratory/Medical Technologists Procedures Manual [35]. Fasting blood samples were collected in K3EDTA vacuum tubes (BD Medical Systems) and analyzed in duplicate within 20 min of collection using Beckman Coulter MAXM/HMX hematology analyzers. Serum albumin was quantified at Collaborative Laboratory Services via the bromcresol purple method on a Beckman Coulter DxC800 analyzer, measuring absorbance at 600 nm. Detailed laboratory methodologies are further documented at: https://wwwn.cdc.gov/nchs/nhanes/continuousnhanes/labmethods.aspx?Cycle=2017-2018.

### Covariates

Our analysis controlled for potential confounders across three categories: (1) population characteristics, including age, gender, ethnicity, marital status, education, and socioeconomic status; (2) behavioral factors, including tobacco and alcohol use; and (3) chronic medical conditions, including cardiovascular disease (CVD), hypertension, and diabetes. These variables were systematically collected using standardized methods, including structured interviews, clinical assessments, and biochemical analyses.

Demographic variables are divided as follows: The race/ethnicity variable is divided into non-Hispanic White, non-Hispanic Black, Mexican American, Other Hispanic, and other. Marital status was classified as “married/cohabiting”, “Unmarried”, “widowed/divorced/separated”. The education variable is divided into below high school, graduation, and above. BMI is classified into three grades based on the clinical line: normal < 25, overweight 25-29.9, and obese ≥ 30. Socioeconomic status was assessed using the family poverty income ratio (PIR). A PIR of ≤ 1.3 indicates low income, 1.3–3.5 indicates medium income, and ≥ 3.5 indicates high income. Living habits can also be classified in detail: If one smokes less than a hundred cigarettes in a lifetime, it is considered as never having smoked; if one smokes more than a hundred and has quit, it is considered as having smoked; if one smokes more than a hundred, it is considered as smoking currently. If one drinks less than 12 cups in a lifetime, it is considered as never having drunk. If one drinks 12 cups but has not touched it in the past year, it is considered as having drunk. If one has drunk in the past year, it is classified by gender: women ≤ 1 cup per week and men ≤ 2 cups per week are considered light; women 2–3 cups and men 3–4 cups are considered medium; women ≥ 3 cups and men ≥ 4 cups are considered heavy. The definition of chronic diseases was based on the test report and self-report: Regarding blood pressure, if the doctor reported high blood pressure, the average systolic/diastolic pressure is ≥ 140/90 mmHg, or if antihypertensive drugs are taken, any one of them indicates hypertension. For blood sugar, if a doctor confirms the diagnosis, hypoglycemic drugs are taken, random blood glucose ≥ 11.1, 2-hour OGTT ≥ 11.1, fasting ≥ 7.0 or HbA1c ≥ 6.5%, any one of these indicates diabetes. For the cardiovascular system, as long as a doctor has reported angina pectoris, heart failure, coronary heart disease, myocardial infarction or stroke, it all counts as CVD. All disease names are determined in accordance with the unified process of NHANES, where the questionnaires, physical examinations, and laboratory results are all matched before the final decision is made.

### Statistical analysis

The entire analysis process followed the complex sampling design, calculating the weights clearly and precisely. Classified data are presented as the number of cases and percentages. Continuous data are presented as the mean ± standard error (SE). The differences between continuous variables were tested using the weighted Student’s t-test, while the cross-group differences of categorical variables were tested using the weighted χ² test.

ALI values were log-transformed to approximate a normal distribution.Then, treat it as a continuous variable and categorized into quartiles. Both the original and logarithmic distributions are presented in the Supplementary Table S1. We constructed three sequential logistic regression models with progressive adjustment for covariates: Model 1 was unadjusted; Model 2 adjusted for demographic factors such as age, race, gender, income, education, and marriage; Model 3 additionally adjusted for clinical and lifestyle factors such as BMI, alcohol consumption, hypertension, diabetes, and CVD. Throw the quartile logarithm ALI as a continuous quantity into the model and conduct a linear trend test. The dose-response curve was visualized using restricted cubic splines (RCS). To verify stability, all covariates are decomposed into subgroup analyses, and interactions are also calculated. Predictivepower is demonstrated by receiver operating characteristic (ROC), with the area under the curve (AUC) as the goldstandard. The DeLong method tested if the fully adjusted model has lifted the curve.

### Sensitivity analysis

To see if the lack of data would lead the conclusion astray, we conducted a few sensitivity tests: First, we singled out those with complete variables for “complete cases”, using them as the yardstick for the main analysis; Second, we retained all participants and grouped the missing items into one category. Finally, multiple imputations are used to extract all the information, and all the results are thrown into the supplementary materials.

### Model development and validation

Among the 37,952 final records, the data was randomly split into 75% for the training set (*n* = 28,464) and 25% for the test set (*n* = 9,488). The complete workflow was designed to ensure a rigorous separation between model development and evaluation phases. To prevent data leakage, all feature preprocessing parameters were learned exclusively from the training set and then applied to the test set. Furthermore, the feature selection process and class imbalance handling were also conducted solely within the training set. To address the prevalent class imbalance, we systematically compared two techniques: ROSE and SMOTE technique. The ROSE method, which generates new synthetic examples based on a smoothed bootstrap approach from the minority class, was empirically found to provide superior and more robust generalization performance across our model ensemble compared to SMOTE’s k-nearest neighbor interpolation. Consequently, superior and more robust generalization performance data was selected for all subsequent model development. Subsequently, the Boruta algorithm, embedded within a random forest framework, was employed on the training set to identify the key risk factors for CRC. This algorithm performs a robust all-relevant feature selection by comparing the importance of original attributes with that of randomly permuted shadow attributes. We trained and validated a total of seven machine learning models: extreme gradient boosting (XGBoost), decision tree (DT), multilayer perceptron (MLP), neural networks (NNET), K-nearest neighbors (KNN), light gradient boosting machine (LightGBM), and support vector machine (SVM), using traditional Logistic Regression as a baseline control. A nested validation approach was implemented. In the inner loop, hyperparameter tuning for each model was performed via a 5-fold cross-validation combined with a grid search strategy on the training set, with the objective of maximizing the mean cross-validation AUC. Key hyperparameters optimized included, but were not limited to, the number of estimators and maximum depth for tree-based models, the regularization parameter (C) for SVM and Logistic Regression, and the learning rate for gradient boosting models. In the outer loop, the final tuned models, with their optimal hyperparameters, were evaluated only once on the held-out test set to provide an unbiased estimate of generalization performance. The models were evaluated using a comprehensive set of metrics: AUC, accuracy, precision, specificity, sensitivity, negative predictive value (NPV), and F1-score. The AUC was prioritized as the primary metric for model selection and comparison due to its robustness to class imbalance. Finally, to ensure explainability, SHAP values were computed on the test set to interpret the output of the optimal model, quantifying the contribution of each feature to individual predictions.

The entire set of operations was completed in R 4.4.1, and the weights of NHANES from 1999 to 2018 were averaged out according to the recommended guidelines with the help of the survey package. The ML part was implemented using the tidymodels framework with the themis package for completion. A bilateral p value < 0.05 is considered to have crossed the significance line.

## Results

### Baseline characteristics of the study participants

The profile of the study population was laid out in Table 1: 37,952 people were shortlisted, with an average age of 47.09 years, among whom 259 were diagnosed with CRC. Notably, CRC patients were significantly older, with a mean age of 68.58 years. Compared with non-CRC participants, CRC prevalence was significantly higher (all *p* < 0.05) among those who were Non-Hispanic White, had a history of former smoking, mild alcohol consumption, or diagnoses of hypertension, diabetes, or CVDs. Marital status also showed a significant association, with higher prevalence in widowed/divorced/separated individuals. Notably, both ALI and log-ALI levels differed significantly between CRC and non-CRC groups (*p* < 0.001), indicating their potential discriminative value.

**Table 1:** Baseline of included participants by the presence of colorectal cancer

Characteristic	Total (*N* = 37,952)	No Colorectal cancer (*N* = 37,693)	Colorectal cancer (*N* = 259)	*P*-value
**Neutrophil count**	4.295(0.017)	4.295(0.017)	4.203(0.123)	0.455
**Lymphocyte count**	2.146(0.010)	2.148(0.010)	1.888(0.067)	< 0.001
**Albumin**,** (g/dl)**	4.284(0.004)	4.284(0.004)	4.164(0.028)	< 0.001
**BMI**,** (kg/m2)**	28.870(0.069)	28.871(0.069)	28.579(0.433)	0.503
**ALI**	68.434(0.540)	68.481(0.542)	59.121(2.537)	< 0.001
**Log-ALI**	4.103(0.004)	4.104(0.004)	3.947(0.035)	< 0.001
**Age**,** years**	47.090(0.210)	46.982(0.210)	68.581(1.127)	< 0.001
**Gender**,** n (%)**				*0.081*
*Female*	*18,835(50.591)*	*18,706(50.559)*	*129(57.110)*	
*Male*	*19,117(49.409)*	*18,987(49.441)*	*130(42.890)*	
**Race**,** n (%)**				*< 0.001*
*Mexican American*	*6472(7.829)*	*6455(7.860)*	*17(1.760)*	
*Non-Hispanic Black*	*7507(10.094)*	*7463(10.106)*	*44(7.817)*	
*Non-Hispanic White*	*17,812(70.661)*	*17,635(70.585)*	*177(85.706)*	
*Other Hispanic*	*2977(5.035)*	*2964(5.048)*	*13(2.444)*	
*Other Race*	*3184(6.380)*	*3176(6.401)*	*8(2.273)*	
**Education level**,** n (%)**				*0.152*
*Below high school*	*9557(15.480)*	*9473(15.452)*	*84(21.109)*	
*High school*	*8779(23.702)*	*8722(23.713)*	*57(21.454)*	
*Above high school*	*19,616(60.818)*	*19,498(60.835)*	*118(57.437)*	
**Poverty level**,** n (%)**				*0.167*
*Low income*	*11,257(20.237)*	*11,183(20.234)*	*74(20.827)*	
*Moderate income*	*14,444(35.674)*	*14,330(35.642)*	*114(42.107)*	
*High income*	*12,251(44.089)*	*12,180(44.124)*	*71(37.066)*	
**Marital status**,** n (%)**				*< 0.001*
*Married/Living with Partner*	*23,093(64.458)*	*22,958(64.506)*	*135(54.962)*	
*Widowed/Divorced/Separated*	*8364(18.290)*	*8251(18.174)*	*113(41.332)*	
*Never married*	*6495(17.253)*	*6484(17.321)*	*11(3.705)*	
**Smoking status**,** n (%)**				*< 0.001*
*Never*	*20,291(53.827)*	*20,186(53.886)*	*105(42.243)*	
*Former*	*9600(25.034)*	*9479(24.940)*	*121(43.591)*	
*Now*	*8061(21.139)*	*8028(21.174)*	*33(14.166)*	
**Alcohol consumption**,** n (%)**				*< 0.001*
*Never*	*5242(10.760)*	*5210(10.755)*	*32(11.833)*	
*Former*	*6598(14.037)*	*6520(13.966)*	*78(27.985)*	
*Mild*	*12,743(36.359)*	*12,631(36.328)*	*112(42.542)*	
*Moderate*	*5742(17.433)*	*5722(17.475)*	*20(9.233)*	
*Heavy*	*7627(21.410)*	*7610(21.476)*	*17(8.406)*	
**Diabetes**,** n (%)**				*< 0.001*
*No*	*32,198(88.762)*	*32,012(88.836)*	*186(74.121)*	
*Yes*	*5754(11.238)*	*5681(11.164)*	*73(25.879)*	
**Hypertension**,** n (%)**				*< 0.001*
*No*	*21,895(62.793)*	*21,835(62.962)*	*60(29.178)*	
*Yes*	*16,057(37.207)*	*15,858(37.038)*	*199(70.822)*	
**CVD**,** n (%)**				*< 0.001*
*No*	*33,880(91.652)*	*33,705(91.763)*	*175(69.765)*	
*Yes*	*4072(8.348)*	*3988(8.237)*	*84(30.235)*	

Note: Mean (SD) for continuous variables: the *P* value was calculated by the weighted Students T-test

Percentages (weighted N, %) for categorical variables: the *P* value was calculated by the weighted chi-square test

Abbreviation: ALI, advanced lung cancer inflammation index; BMI, body mass index; CVD, cardiovascular disease

### Correlation between ALI and CRC risk

**Fig. 2:**
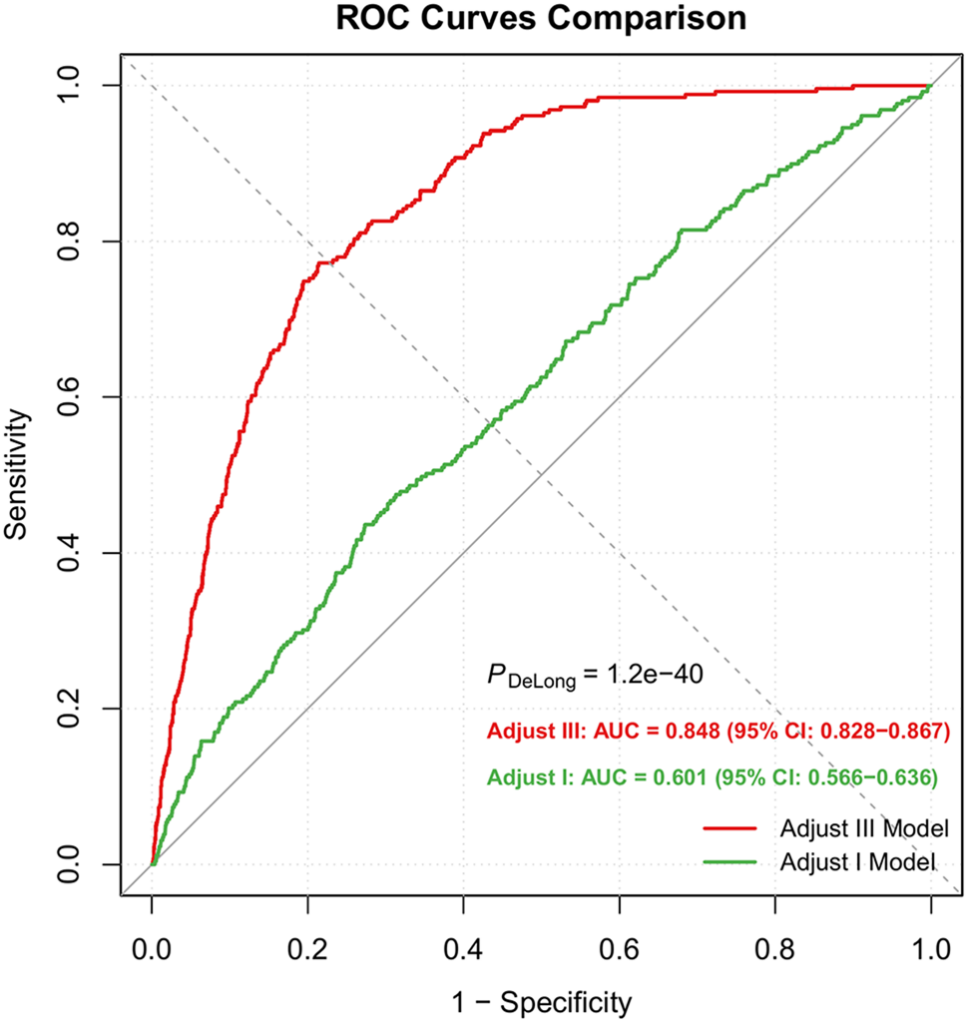
Receiver operating characteristic (ROC) between log-ALI and colorectal cancer in Model 1 and Model 3

**Fig. 3:**
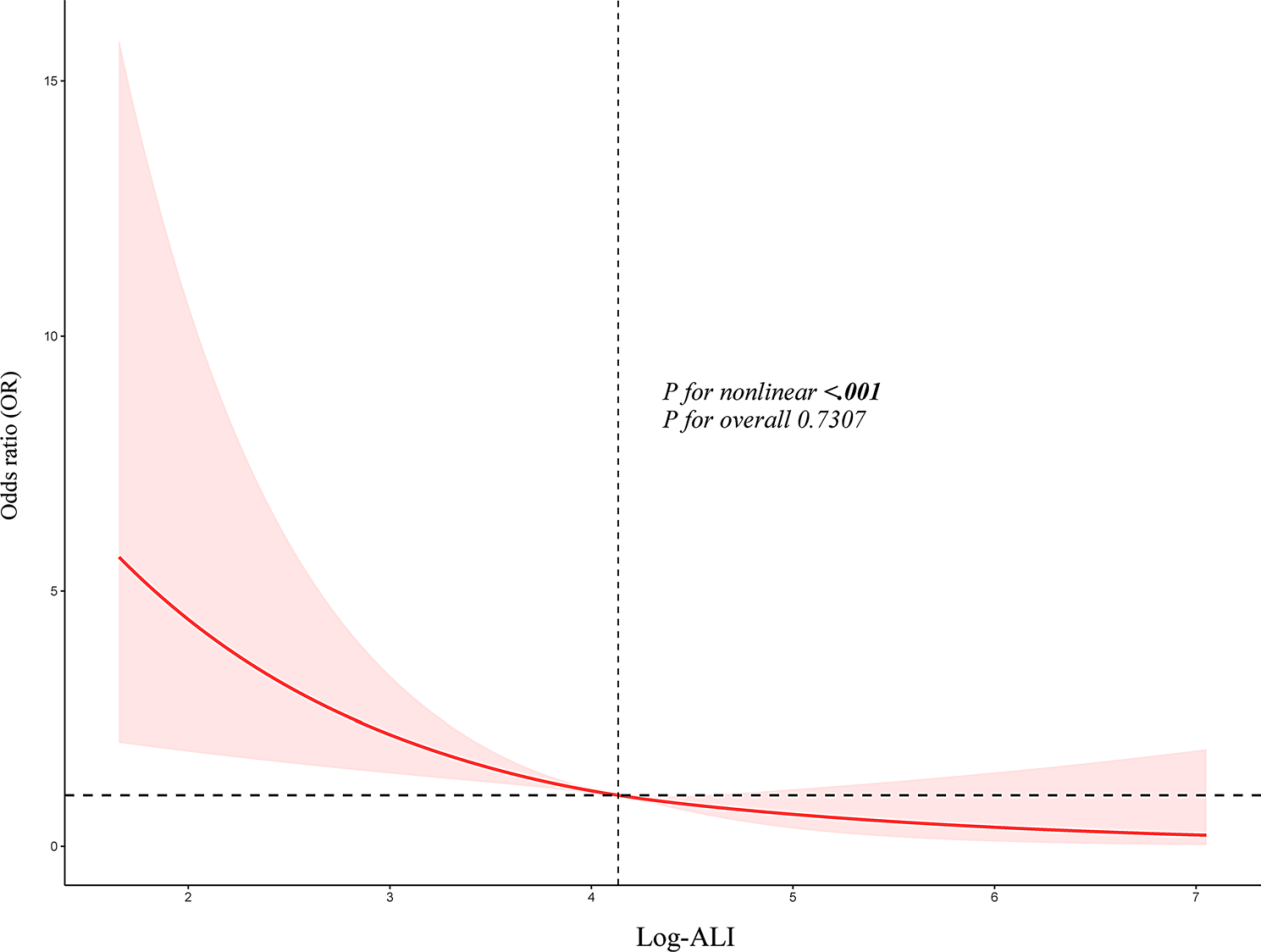
The dose - response relationship between log-ALI level and colorectal cancer risk. The model has been adjusted for age, race, gender, poverty level, education, marital status, BMI, smoking status, alcohol drinking, hypertension, diabetes, angina, stroke, and CVD

First, take the logarithm of ALI, and then layer it into the model by continuous quantity and quartile simultaneously. The association between log-ALI and CRC risk was assessed using logistic regression. The results are listed in the Table 2. In the unadjusted model (Model 1), for each unit increase in log-ALI, the CRC probability dropped by 47.6% (OR = 0.524; 95% CI: 0.400-0.686; *P* < 0.001). After adjustment for age, race, gender, poverty level, education, and marital status in Model 2, the negative association still persisted, with an OR of 0.771 (95% CI: 0.606–0.980; *P* = 0.034). In Model 3, BMI, smoking, drinking, hypertension, diabetes and CVD were further included. The results remained the same: for each increase in log-ALI, the risk of CRC decreased by more than 20%, with an OR of 0.791 (95% CI: 0.628–0.997; *P* = 0.047). In accordance with contemporary statistical guidelines, we interpret this finding as indicative of a statistically significanttrend. In the quartile analysis, compared with the bottom-ranked Q1, Model 1 showed a 64.9% reduction in the risk for the topmost Q4 (OR = 0.351; 95% CI: 0.226–0.545; *P* < 0.001). After all covariates were adjusted, the decline remained at nearly 46.2% (OR = 0.538; 95% CI: 0.344–0.842; *P* = 0.007). After maximizing all covariates, the model’s AUC soared to 0.848(95% CI:0.828–0.867), outperforming the unadjusted version. The DeLong test also signed a *p* < 0.001 (Fig. 2). RCS analysis confirmed a linear dose-response relationship between log-ALI and CRC risk (P for nonlinearity = 0.731)(Fig. 3). The results remained consistent in sensitivity analyses that excluded extreme ALI values and in analyses performed both before and after multiple imputation (Supplementary Table S1-S3).

**Table 2:** Association between advanced lung cancer inflammation index levels and colorectal cancer among US participants, NHANES, 1999 to 2018

ALI	Model I [OR (95% CI)]	*p*-value	Model II [OR (95% CI)]	*p*-value	Model III [OR (95% CI)]	*p*-value
Per ln-unit increase	0.524(0.400,0.686)	**< 0.001**	0.771(0.606,0.980)	**0.034**	0.791(0.628,0.997)	**0.047**
Q1	Reference		Reference		Reference	
Q2	0.703(0.435,1.135)	0.148	0.949(0.584,1.543)	0.832	0.969(0.601,1.561)	0.896
Q3	0.704(0.472,1.050)	0.085	1.041(0.700,1.549)	0.840	1.077(0.731,1.585)	0.706
Q4	0.351(0.226,0.545)	**< 0.001**	0.534(0.340,0.838)	**0.007**	0.538(0.344,0.842)	**0.007**
*P for trend*		**< 0.001**		**0.041**		**0.044**

Note: Abbreviation: ALI: advanced lung cancer inflammation index; NHANES, National Health and Nutrition Examination Survey; BMI, Body mass index; CVD, cardiovascular disease. OR, odds ratio; CI, confidence interval

Model I was unadjusted

Model II was adjusted for age, race, gender, poverty level, education and marital status

Model III was adjusted for age, race, gender, poverty level, education, marital status, BMI, smoking status, alcohol consumption, hypertension, diabetes, and CVD

### Subgroup analyses

**Fig. 4:**
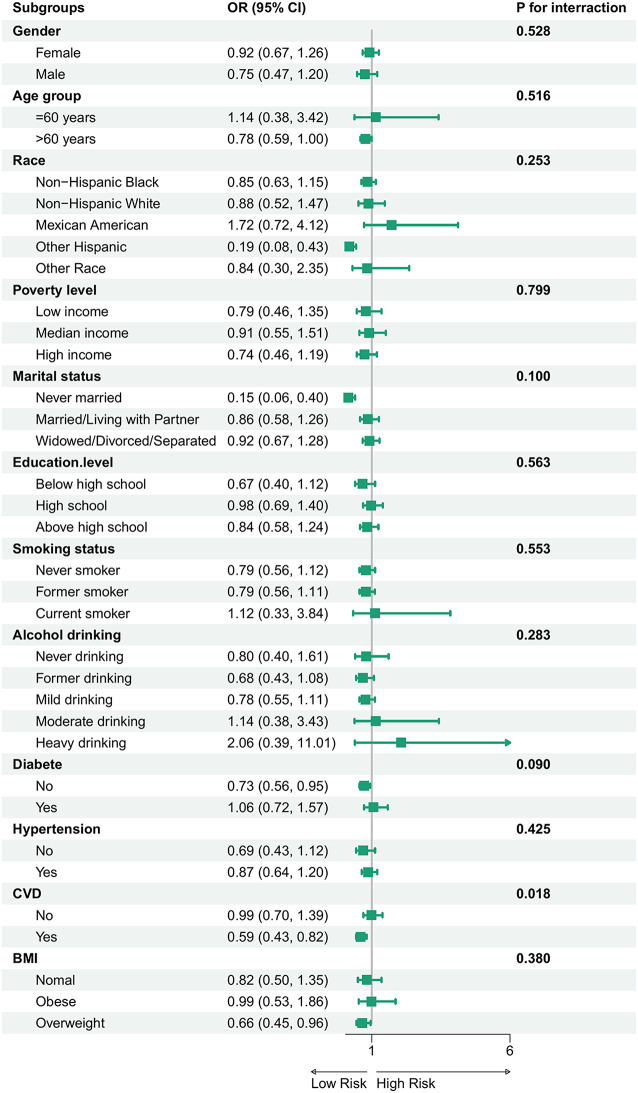
Subgroup analyses of the association between log-ALI level and colorectal cancer. Notes: ALI, advanced lung cancer inflammation index. Analyses were adjusted for age, race, gender, poverty level, education, marital status, BMI, smoking status, alcohol drinking, hypertension, diabetes, angina, stroke, and CVD

Subgroup analyses were performed to assess the association between log-ALI and CRC across various population strata (Fig. 4). The inverse association between log-ALI and CRC was stronger in the elderly (> 60 years old), non-Hispanic whites, obese individuals with BMI ≥ 30, unmarried individuals, those without diabetes, and those with CVD. Among them, the interaction between CVD and ALI reached *P* = 0.018, with the most significant difference.

### Random forest analysis

**Fig. 5:**
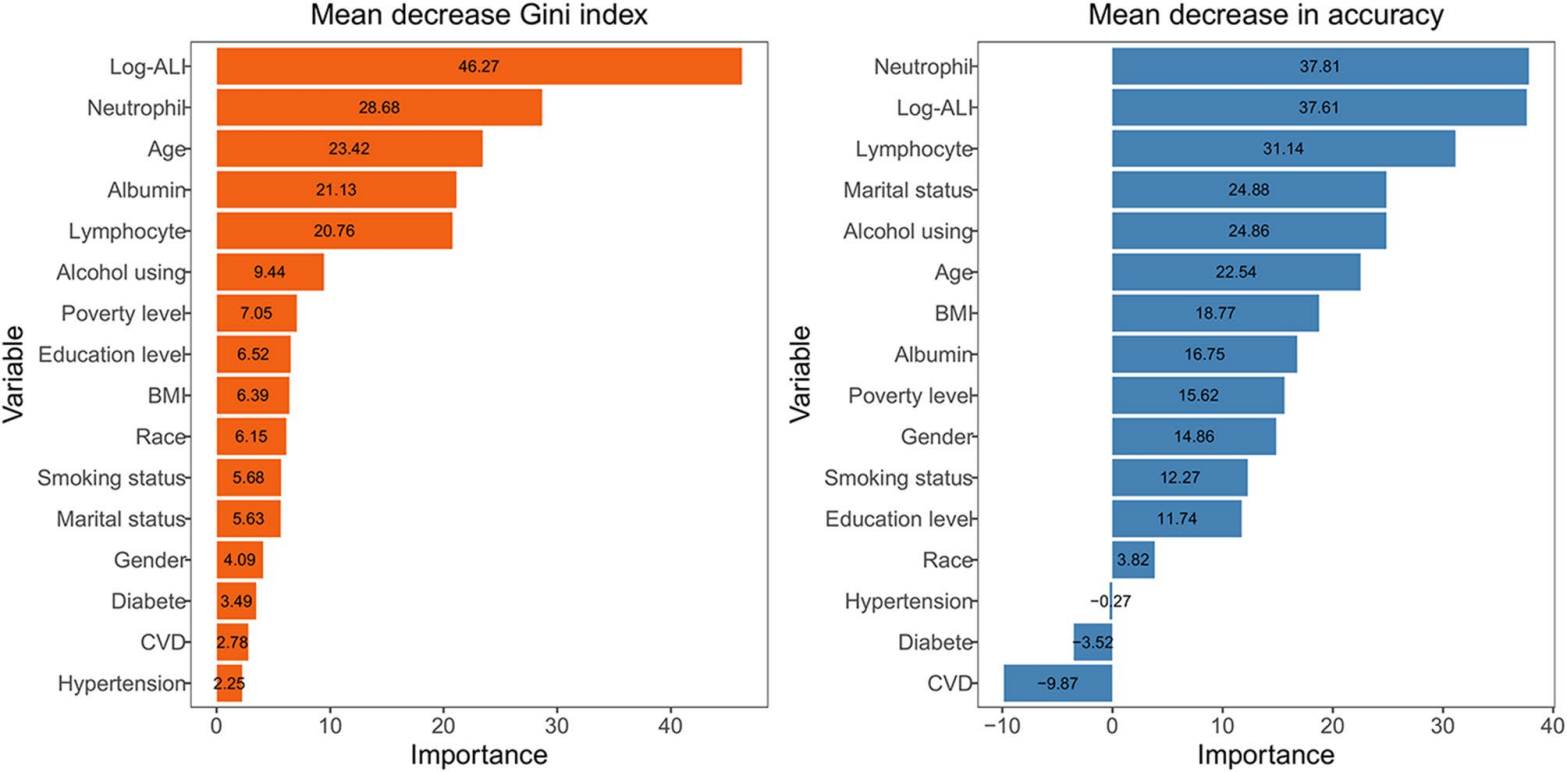
Variable importance according to the random forest analysis, evaluated by mean decrease in the Gini index (**a**) and mean decrease in accuracy (**b**). Notes: ALI, advanced lung cancer inflammation index; BMI, body mass index; CVD, cardiovascular disease

To identify key determinants of CRC risk, we applied random forest modeling incorporating all covariates from the fully adjusted model. As shown in Fig. 5, feature importance visualization indicates that higher values of both mean decrease in Gini index and mean decrease in accuracy correspond to greater predictive significance. Notably, based on the mean decrease in the Gini index, log-ALI was identified as the most important predictor. It also ranked as the second most influential variable when assessed by the mean decrease in accuracy, confirming its robust and independent predictive value for CRC.

### Boruta algorithm

**Fig. 6:**
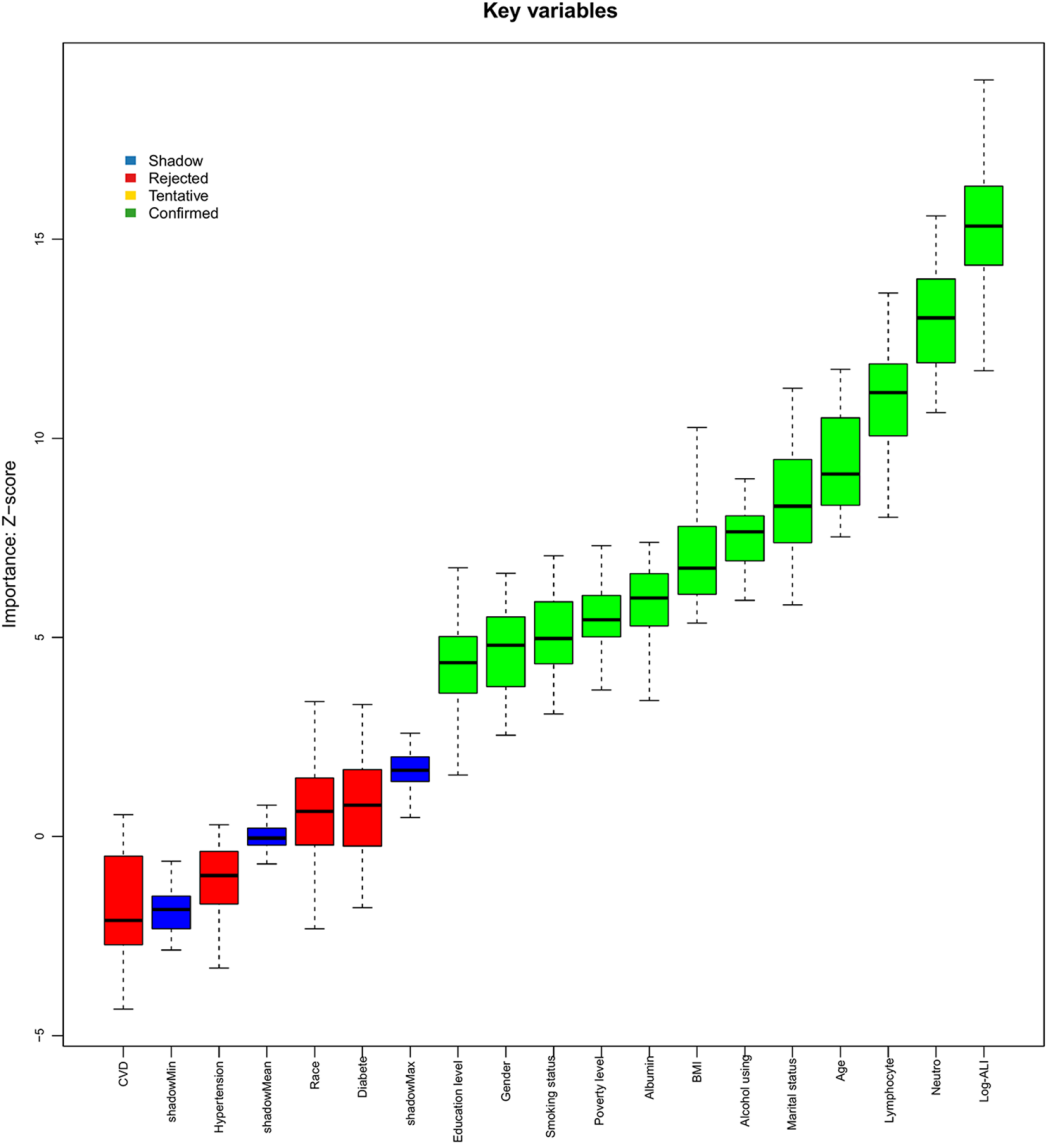
Predictor importance for angina pectoris according to the Boruta algorithm. A predictor was deemed important if its mean importance Z-score was significantly higher than the maximum value of the shadow variables (blue). Conversely, a predictor (red) was excluded if its mean importance Z-score was significantly lower than the maximum value of the shadow variables. Notes: ALI, advanced lung cancer inflammation index; BMI, body mass index; CVD, cardiovascular disease

Figure 6 presents the results of the Boruta algorithm, which identified key features associated with CRC. Variables located in the green zone were classified as important predictors. After 500 iterations, 12 significant variables were retained, including log-ALI, neutrophil, lymphocyte, age, marital status, alcohol consumption, BMI, albumin, poverty level, smoking status, sex and education level. The results show that the weight of logarithmic ALI has outperformed other indicators and firmly holds the top position. For final model development, diabetes, hypertension, CVD, and race were purposefully included due to their clinical relevance.

### ML predictive models and representative interpretations

**Fig. 7:**
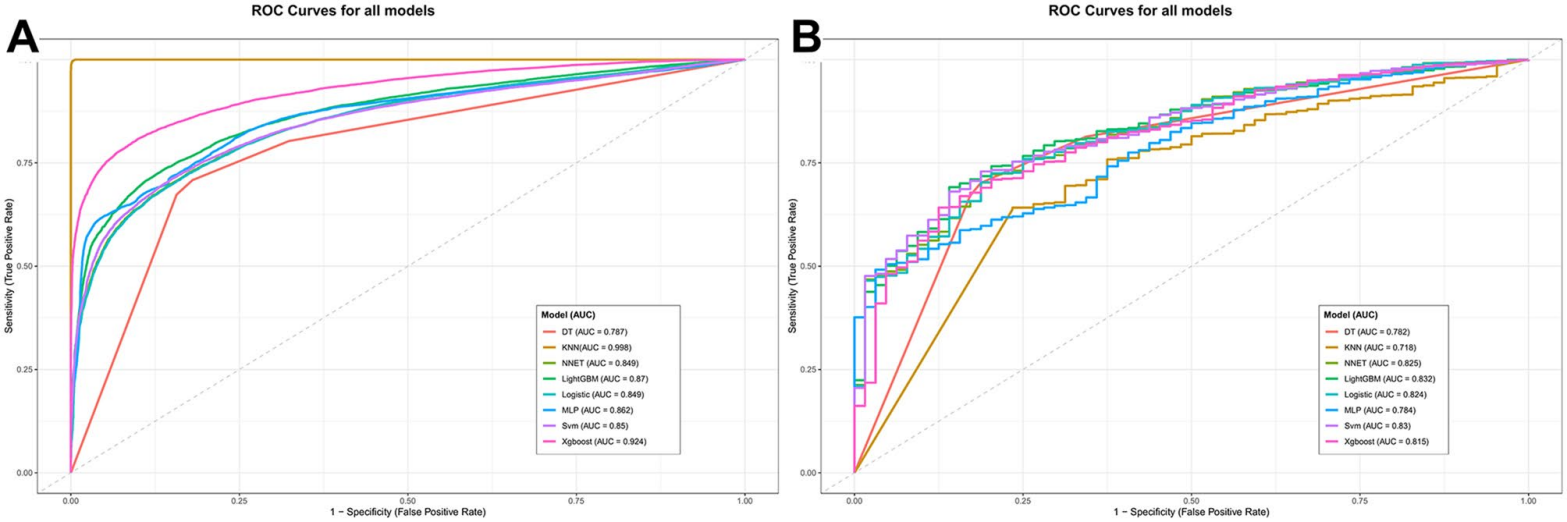
Receiver operating characteristic (ROC) curves of the eight machine-learning models.To evaluate the performance of the different ML methods, we compared eight ML algorithms: XGBoost, DT, MLP, NNET, KNN, LightGBM, SVM and logistic model Fig. 7. (**A**) ROC curves of the training set. (**B**) ROC curves of the testing set

To address class imbalance, both the ROSE and SMOTE algorithms were evaluated. Comparative analysis revealed that the ROSE method provided superior and more robust generalization performance across the ensemble of models. Consequently, the ROSE-processed dataset was selected for all subsequent analyses, while results based on SMOTE are provided in the Supplementary Figure S2 and Supplementary Table S4. The predictive performance of eight distinct algorithms was evaluated and compared. (Fig. 7). When comparing the training set with the test set, LightGBM maintained a consistent lead, remaining stable without any fluctuations. The AUC of the test set reached 0.832, the highest in the entire competition, while also maintaining strong performance on the training set (AUC: 0.870), reflecting a well-balanced learning capacity and maintaining minimal overfitting. The model also exhibited excellent precision (0.999), specificity (0.860), and F1-score (0.817), underscoring its clinical reliability. Furthermore, LightGBM outperformed the logistic regression model, which attained AUC values of 0.848 on the training set and 0.824 on the test set. The model’s robustness was further validated through 5-fold cross-validation, demonstrating consistent performance improvement throughout the tuning process (Supplemental Figure S3). A comprehensive comparison of all evaluated models is provided in Supplementary Table S5.

To contextualize these results, we compared our model against several established CRC risk assessment models derived from the NHANES database, including inflammation-based prognostic markers. As shown in Supplemental Figure S4 and nd Supplementary Table S6 our model achieved a higher AUC value(0.940), indicating superior predictive performance relative to these existing biomarkers. We further assessed the performance of the LightGBM model across key clinical subgroups. As summarized in Supplemental Figure S5 and Supplementary Table S7, the model consistently demonstrated strong discriminative ability. As detailed in Supplementary Table S7, the model demonstrated strong and consistent discriminative ability (AUC > 0.84) in several major subgroups, including older adults (> 60 years) and non-diabetic participants.

**Fig. 8:**
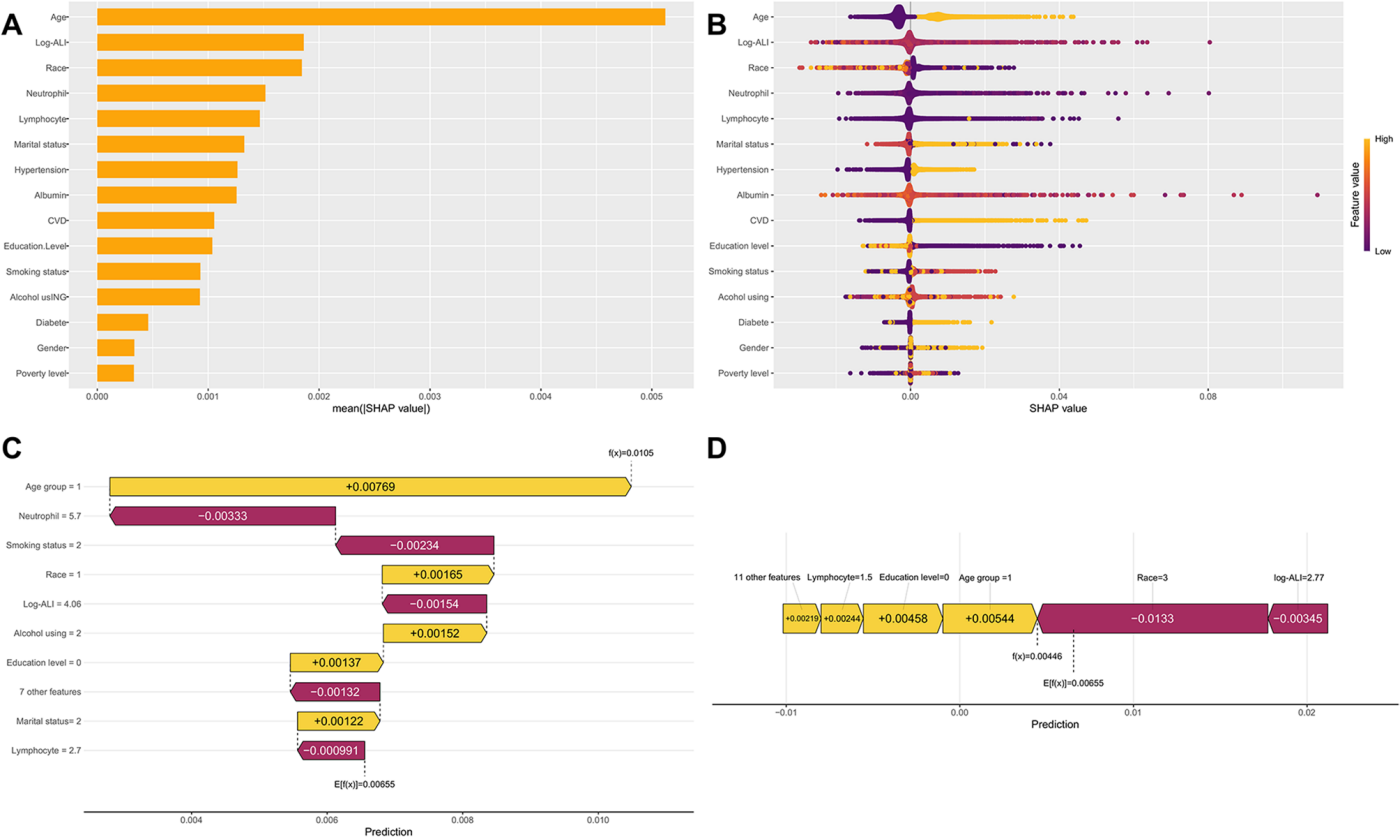
SHAP diagram of LightGBM model. (**A**) SHAP value ranking of the variables. (**B**) SHAP honeycomb diagram of the LightGBM model. (**C**) SHAP waterfall plot. (**D**) Shap force plot

To evaluate the contribution of individual predictors to CRC risk estimation within the LightGBM model, we performed SHAP analysis. This method quantifies the importance of each feature and its directional impact on model predictions (Fig. 8A and B). Among the 15 predictors analyzed, log-ALI exhibited the highest mean absolute SHAP value. This confirms its role as the most influential protective factor against CRC. The finding aligns with prior clinical expectations. To further enhance model interpretability, we generated SHAP waterfall plots and force plot illustrating the direction and magnitude of feature contributions to the prediction for individual participants, as exemplified by the second participant in the study cohort (Fig. 8C and D).

## Discussion

As the first nationally representative study examining the association between ALI and CRC, multivariable regression and RCS analyses confirmed a linear inverse relationship. The robustness of this finding was supported by subgroup and sensitivity analyses. ROC analysis demonstrated the strong discriminative ability of ALI for CRC prevalence. Feature selection using random forest and the Boruta algorithm further emphasized the diagnostic relevance of ALI. To enhance predictive accuracy, seven machine learning models were employed, with SHAP values providing interpretable insights into feature contributions. Collectively, the results highlight the pivotal clinical value of ALI in CRC risk assessment. This study establishes ALI as a protective factor against CRC and suggests that its risk prediction model may offer practical utility for CRC prevention and early intervention.

Compared with existing CRC risk assessment tools, the ALI-based model developed in this study offers several distinct advantages. While the Asia-Pacific Colorectal Screening (APCS) score demonstrates reasonable clinical utility, it relies exclusively on basic demographic features such as age, sex, and family history [36]. In contrast, the ALI model incorporates objective biomarkers, enabling a more biologically informed risk assessment. Similarly, although the Harvard Cancer Risk Index covers a wider range of lifestyle factors [37], it depends on self-reported data that are susceptible to recall bias. Genetic-based prediction models perform well in specific populations [38], yet their broader applicability is constrained by high testing costs. In terms of predictive performance, a large-scale external validation in the UK Biobank indicated that the best existing models achieved an AUC of 0.67–0.70 in males, with even lower values in females [39]. By comparison, the ALI model not only exhibits superior discriminative ability but may also be less affected by population heterogeneity, as it utilizes routinely available and objective clinical measures. From a clinical implementation perspective, the ALI model has the potential to enhance current CRC screening pathways. Although colonoscopy remains the gold standard [40], its invasiveness limits widespread repeated use. Fecal immunochemical testing, the most widely used non-invasive screening method for average-risk individuals [41], could be strategically combined with the ALI model to improve follow-up adherence in screening programs [42]. Emerging multitarget stool DNA tests provide high sensitivity but are restricted by cost and accessibility [42]. Furthermore, by offering risk stratification rather than a binary outcome, the ALI model may help resolve clinical ambiguity in cases where multitarget stool DNA tests are negative yet colonoscopy results remain inconclusive [43]. Compared to traditional serum biomarkers (such as CEA and CA199), which are limited in early detection, the ALI model utilizes routine clinical parameters without necessitating additional assays. Advanced liquid biopsy techniques, including circulating tumor cells [44] and circulating tumor DNA [45], represent promising alternatives; however, their technical complexity and high cost currently hinder broad adoption in population-wide screening.

CRC development and progression result from the interplay of genetic susceptibility and environmental factors, with chronic inflammation, oxidative stress, and gut microbiota dysbiosis representing core pathogenic mechanisms [46, 47]. Persistent reactive oxygen species (ROS) accumulation may induce critical mutations in tumor suppressor genes and proto-oncogenes, thereby driving tumorigenesis [48]. Chronic inflammation compromises intestinal mucosal barrier integrity, impairs host defense, and promotes pathogen invasion, creating a self-perpetuating cycle [49]. Substantial clinical evidence indicates that chronic inflammation frequently precedes or accompanies tumor progression [50]. In this context, ALI represents a quantifiable biomarker warranting further investigation in tumor-associated inflammatory processes. Chronic inflammation promotes carcinogenesis through multiple mechanisms. Sustained inflammatory responses generate ROS, exacerbating oxidative damage [51]. In CRC, intensified inflammation reshapes the composition of epithelium-adherent microbiota, favoring species that harbor genotoxic gene products. For example, certain Escherichia coli strains produce colibactin, which induces host cell mutations [52]. Upon recognition by myeloid cells, translocated microbial metabolites amplify tumor-associated inflammation through IL-23-dependent pathways and related mechanisms [53]. Additionally, macrophage-derived factors, such as TGF-β, contribute to the formation of an immunosuppressive tumor microenvironment [54], ultimately enhancing cancer stemness and promoting immune evasion [55]. Nutritional status plays a critical role in both the initiation and progression of CRC. Epidemiological evidence indicates that obesity is a significant risk factor, with individuals exhibiting a BMI >30 kg/m² showing a 1.5- to 1.8-fold increased CRC risk [56]. Obesity contributes to CRC through multiple mechanisms. Visceral adipose tissue secretes adipokines, including leptin and resistin, which activate key oncogenic pathways [57]. Obesity is also frequently associated with insulin resistance and hyperinsulinemia, whereby insulin and related growth factors promote tumor growth by enhancing cellular proliferation and inhibiting apoptosis [58, 59]. Additionally, obesity induces chronic low-grade inflammation, characterized by elevated pro-inflammatory cytokine production and persistent systemic inflammatory responses [60]. Conversely, malnutrition is associated with increased CRC susceptibility. Deficiencies, including hypoalbuminemia and sarcopenia, progressively impair immune function, notably reducing immune surveillance against abnormal cells [61], thereby creating a permissive environment for tumor initiation and progression.

The ALI integrates three key parameters: BMI, circulating albumin, and NLR. BMI and albumin serve as nutritional indicators, whereas NLR reflects systemic inflammation, rendering ALI a holistic biomarker that simultaneously captures metabolic and inflammatory status. Neutrophils, which constitute 50–70% of circulating leukocytes, play multifaceted and often contradictory roles in CRC progression [62]. They contribute to tumor development through the release of IL-1β [63], yet also secrete CCL17 to recruit regulatory T cells, facilitating immune evasion [64], and support angiogenesis [65]. Tumor-associated neutrophils (TANs) exhibit considerable plasticity, shifting from anti-tumor to pro-tumor phenotypes [66]. Pro-tumor TANs display altered metabolic activity and secrete factors that promote angiogenesis, tumor growth, and metastasis—such as AGR2, which enhances the metastatic potential of CRC cells [67, 68]. Additionally, neutrophils form neutrophil extracellular traps (NETs) via NETosis [69], which have been linked to circulating tumor cell survival, metastasis, therapy resistance, and poor responses to immunotherapy [70, 71]. Lymphocytes play a central role in antitumor immunity, suppressing tumor proliferation and metastasis [72]. Clinically, peripheral lymphocyte counts serve as reliable prognostic biomarkers, with higher levels generally reflecting stronger antitumor responses mediated by cytotoxic T cells and natural killer cells [73]. Beyond direct cytotoxicity, lymphocytes modulate the tumor microenvironment through cytokine signaling, promoting antigen presentation and helping reverse immunosuppression [74]. The correlation between peripheral and tumor-infiltrating lymphocytes further supports their clinical relevance. Conversely, lymphopenia impairs immune surveillance and promotes tumor immune evasion. Obesity is recognized as a probable risk factor for CRC by leading international agencies [75]. Adipose tissue, particularly visceral fat, secretes pro-inflammatory cytokines, which contribute to tumorigenesis [76]. Adipose tissue inflammation also promotes insulin resistance and hyperinsulinemia, with insulin and insulin-like growth factors exerting mitogenic and anti-apoptotic effects [77]. Additionally, adipocytes release adipokines including leptin, which enhances cellular proliferation and invasiveness [78]. Obesity may further drive CRC through epigenetic modifications and alterations in sex hormone levels [79, 80]. Serum albumin serves as an objective biomarker of both nutritional status and systemic inflammation [81]. Low albumin levels are linked to impaired energy metabolism, reduced tolerance to antitumor therapies, and poor clinical outcomes [82]. Albumin exerts protective effects through its antioxidant and immunomodulatory properties, including regulation of antigen presentation and cytokine secretion [83]. Dysregulated albumin levels may promote colorectal carcinogenesis by disrupting redox balance and immune function, while tumor progression itself can further deplete systemic albumin [84]. The ALI index integrates BMI, serum albumin, and the NLR to capture key pathophysiological processes in CRC. Hypoalbuminemia reflects impaired antioxidant and barrier defenses, elevated NLR indicates pro-tumor inflammation, and increased BMI contributes to chronic inflammation and insulin resistance. Together, these components synergistically promote carcinogenesis through sustained oxidative stress, compromised immune surveillance, and enhanced proliferative signaling. As a composite biomarker, the ALI quantitatively encapsulates this multidimensional pathophysiology and shows meaningful correlation with CRC progression and treatment outcomes.

In our subgroup analyses, the inverse association between ALI and CRC exhibited significant interactions in specific populations, suggesting heterogeneous effects of lifestyle and metabolic factors across populations. The enhanced protective effect of ALI in the elderly may be understood within the inflammaging framework, an age-related state of chronic low-grade inflammation and immunosenescence. The attenuated protection in diabetic patients may reflect altered ALI component biology under hyperglycemic conditions. Chronic hyperglycemia promotes tumor proliferation while impairing immune surveillance [77], and induces insulin resistance and albumin glycation that may reduce its antioxidant capacity. The obesity-diabetes comorbidity [85] creates a complex metabolic environment where ALI’s nutritional-inflammatory balance indicators may be compromised. Chronic inflammation serves as a critical link between CVD and cancer development. Patients with CVD often exhibit persistent systemic inflammation, which may facilitate cancer cell proliferation and metastasis. Furthermore, CVD and cancer share several common risk factors, including obesity and hyperglycemia, which collectively contribute to the overlapping pathophysiological processes of both conditions. Concerning marital status, evidence indicates that being unmarried may influence cancer risk through psychosocial mechanisms [86]. Insufficient social support and chronic psychological stress can induce neuroendocrine dysregulation, impair immune surveillance [87].

Our study, utilizing a nationally representative sample, establishes a significant inverse association between ALI and CRC risk. A key methodological strength lies in the integration of complementary analytical approaches, including weighted multivariable logistic regression, RCS, and notable ML algorithms. As a composite biomarker derived from routinely available parameters, ALI is a cost-effective and accessible metric that holds potential for seamless integration into routine clinical practice. Several methodological limitations merit consideration. First, although our analysis incorporated ten cross-sectional surveys and adjusted for multiple covariates, the non-sequential design of this study is a key limitation, as it prevents any determination of causality. Additionally, this inherent limitation of the study design is further compounded by potential residual confounding from unmeasured or imperfectly measured factors, such as detailed dietary patterns, physical activity levels, and other lifestyle variables not comprehensively captured in the NHANES database. We strongly emphasize that future prospective cohort studies with serial ALI measurements are essential to validate these findings and elucidate temporal relationships. Additionally, Mendelian randomization analyses could provide valuable complementary evidence to assess potential causal mechanisms while accounting for unmeasured confounding. Second, CRC case identification relied entirely on self-report, as NHANES lacks imaging or histopathological confirmation. This may affect the accuracy of observed associations. Additionally, the absence of clinical validation limited access to tumor characteristics, constraining detailed subgroup analyses. It should be noted, however, that previous validation studies have demonstrated the reliability of self-reported cancer data, showing high sensitivity for colon cancer and an overall positive predictive value of 0.75 for all cancers combined [88]. Moreover, potential non-differential misclassification of the novel ALI index would likely attenuate the observed effect sizes, indicating that the true association may be stronger than reported. Third, the relatively limited number of CRC cases may constrain the stability of ML models and the detection of subtle predictive patterns, despite our use of algorithms designed for imbalanced data and cross-validation techniques. However, our comprehensive approach, including the use of algorithms specifically designed for imbalanced data, rigorous cross-validation, and independent test set evaluation, provides reasonable assurance of model reliability. The consistent performance observed between training and test sets across multiple algorithms further supports the robustness of our findings. Future validation in larger, independent cohorts will be essential to confirm the generalizability of the ALI-based risk stratification model. Fourth, inherent limitations in laboratory measurements and biological variability should be considered. Although NHANES laboratories maintain CLIA certification and implement rigorous quality control procedures such as standardized protocols, regular instrument calibration, and blinded repeat testing, minor systematic errors between batches may still occur. Additionally, the biological interpretation of ALI components requires caution: serum albumin levels may be influenced by acute conditions and hydration status, and BMI serves only as an approximate indicator of body composition, subject to variation based on measurement timing and specific conditions. We emphasize that the limitations acknowledged in this study do not undermine the reliability of the current results but instead highlight key areas for methodological refinement in future investigations.

This study demonstrates that the ALI-based CRC risk stratification model holds substantial potential for clinical implementation. By utilizing routinely available clinical parameters, the model requires no specialized equipment or additional testing, offering significant advantages in cost-effectiveness and accessibility. The ALI-based risk stratification tool presents a practical approach for enhancing current CRC screening protocols. Its implementation can be achieved through two complementary pathways: integration into hospital laboratory information systems for automated risk calculation using routine parameters (BMI, albumin, and NLR), and development of web-based calculators for primary care settings. This dual approach would enable identification of high-risk individuals for prioritized colonoscopy referral while allowing dynamic monitoring through serial ALI measurements in moderate-risk cases. Several challenges require addressing before widespread implementation. The model necessitates validation across diverse populations and healthcare settings. Successful integration will require appropriate IT infrastructure, staff training, and workflow adjustments, with ALI scores always interpreted within a comprehensive clinical assessment. Looking forward, the predictive performance of the ALI index could potentially be augmented by integrating it with other emerging biomarkers. Particularly promising is the incorporation of gut microbiota signatures, given their established link to both intestinal inflammation and CRC carcinogenesis [89–91]. Future studies that combine inexpensive systemic indices like ALI with multi-omics approaches, including microbiome profiling, are warranted to build more comprehensive and powerful risk stratification tools.

This study thus establishes a foundation for developing more efficient, cost-effective, and precise CRC risk assessment tools that can be readily integrated into diverse healthcare settings, ultimately contributing to improved early detection and prevention of CRC.

## Conclusion

Our study demonstrates a significant inverse association between ALI and CRC susceptibility in American adults. The CRC risk prediction framework incorporating ALI, developed using multiple ML approaches, exhibited robust discriminative performance. These findings underscore the potential protective effects of maintaining optimal nutritional status and modulating systemic inflammation in CRC prevention. We recommend future prospective cohort studies and mechanistic investigations to validate the prognostic utility of ALI for CRC risk stratification.

## Supplementary Information

Below is the link to the electronic supplementary material.

Supplementary Material 1

Supplementary Material 2

Supplementary Material 3

## Data Availability

The datasets used during the current study are available from the NHANES database(https://www.cdc.gov/nchs/nhanes/about/index.html). The datasets used and/or analysed during the current study are available from the corresponding author on reasonable request.

## Abbreviations

NHANES: National Health and Nutrition Examination Survey
CRC: Colorectal cancer
ALI: Advanced lung cancer inflammation index
PIR: Poverty income ratio
RCS: Restricted cubic spline
ROC: Receiver operating characteristic
AUC: Area under the curve
ML: Machine learning
SHAP: Shapley additive explanations
WHO: World Health Organization
NLR: neutrophil-to-lymphocyte ratio
BMI: Body mass index
B2B: Bench-to-bedside
CDC: Centers for disease control and prevention
NCHS: National center for health statistics
MCQ: Medical conditions questionnaire
CVD: Cardiovascular disease
SE: Standard error
XGBoost: Extreme gradient boosting
DT: Decision tree
MLP: Multilayer perceptron
NNET: Neural networks
KNN: K-nearest neighbors
SVM: Support vector machine
LightGBM: Light gradient boosting machine
NPV: Negative predictive value
IARC: International Agency for Research on Cancer
AICR: American Institute for Cancer Research
ctDNA: Circulating tumor DNA
scRNA-seq: Single-cell RNA sequencing
CEA: Carcinoembryonic antigen
ROS: Reactive oxygen species
TANs: Tumor-associated neutrophils
