# Supplementary material for: From biomarker to clinical utility: translating the advanced lung cancer inflammation index into a machine learning-driven risk stratification tool for colorectal cancer
Source: J Transl Med. 2025 Nov 26;24:7. doi: 10.1186/s12967-025-07494-z (PMC12764094; doi:10.1186/s12967-025-07494-z)
Supplement: Supplementary file 1 — Supplementary Material 1 [file 12967_2025_7494_MOESM1_ESM.docx]

Supplementary Material

# Supplementary Figures and Tables

**Supplementary Figure S1.** The distributions of both raw and log-transformed ALI.

**Supplementary Figure S2.** Receiver operating characteristic (ROC) curves of the eight machine-learning models using SMOTE balancing technique. To evaluate the performance of the different ML methods, we compared eight ML algorithms: XGBoost, DT, MLP, NNET, KNN, LightGBM, SVM and logistic model Figure 7 . (A) ROC curves of the training set. (B) ROC curves of the testing set.

**Supplemental Figure S3.** Receiver operating characteristic (ROC) curves showing the performance of the LightGBM(by five-fold cross-validation).

**Supplemental Figure S4.** Performance comparison of our LightGBM model with other existing models.

**Supplemental Figure S5.** Performance comparison of our LightGBM model in different subgroups.

**Supplementary Table S1:** Association between Log-ALI and colorectal cancer by including the NHANES with missing data not handling.

**Supplementary Table S2:** Association between Log-ALI and colorectal cancer by including the NHANES after imputation.

**Supplementary Table S3:** Association between Log-ALI and colorectal cancer by excluding the lower 2.5% and upper 97.5% ALI value.

**Supplementary Table S4.** Performance metrics for 8 models in the train and test datasets using SMOTE balancing technique..

**Supplementary Table S5.** Performance metrics for 8 models in the train and test datasets using ROSE balancing technique.

**Supplementary Table S6.** Performance metrics of our LightGBM model in different subgroups.

**Supplementary Table S7.** Performance metrics of our LightGBM model in different subgroups.

# **Supplementary Figure S1. The distributions of both raw and log-transformed ALI.**


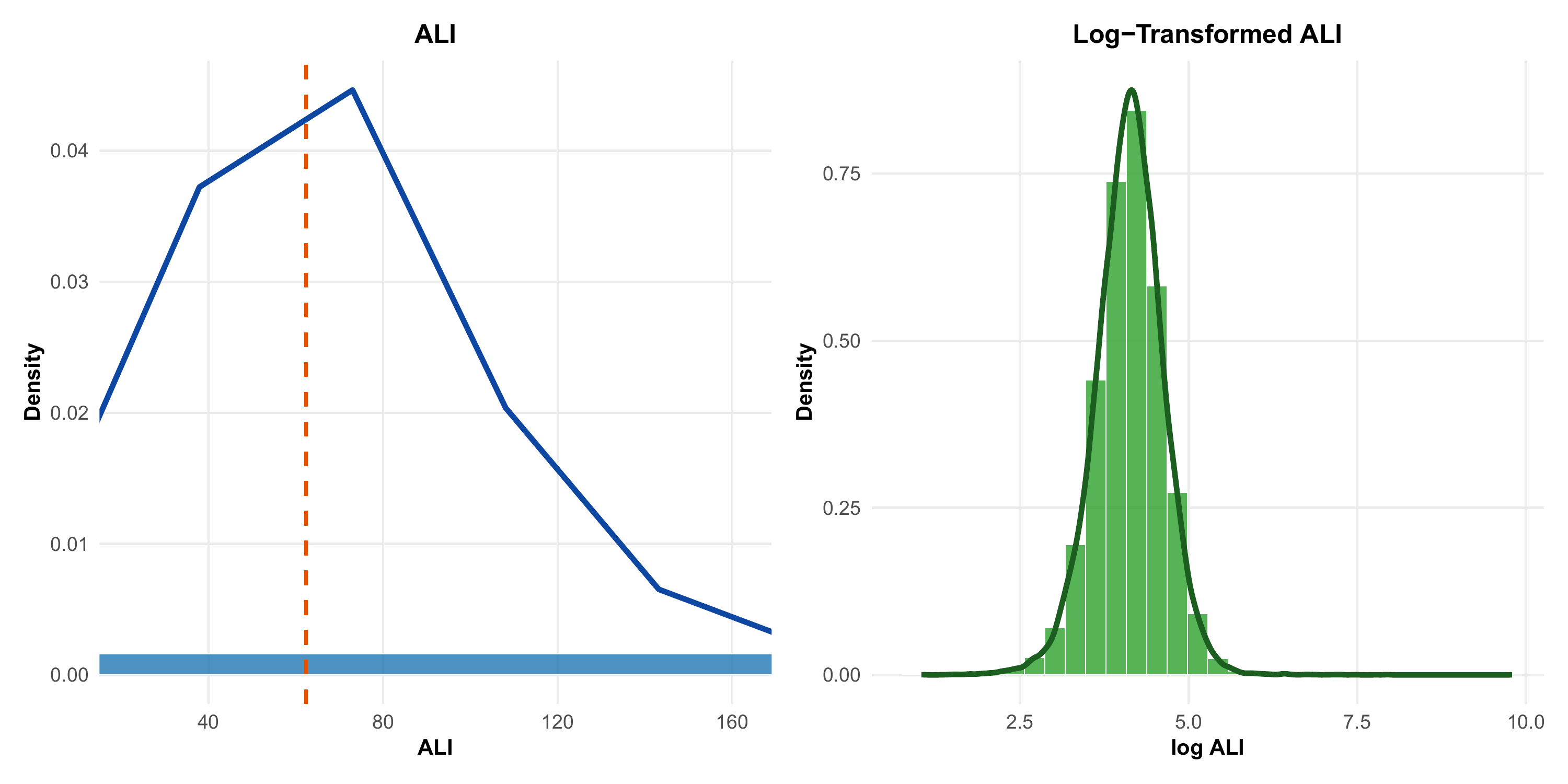
Abbreviation: ALI, advanced lung cancer inflammation index.

**Supplementary Figure S2. Receiver operating characteristic (ROC) curves of the eight machine-learning models using SMOTE method.To evaluate the performance of the different ML methods, we compared eight ML algorithms: XGBoost, DT, MLP, NNET, KNN, LightGBM, SVM and logistic model Figure 7 . (A) ROC curves of the training set. (B) ROC curves of the testing set.**

**
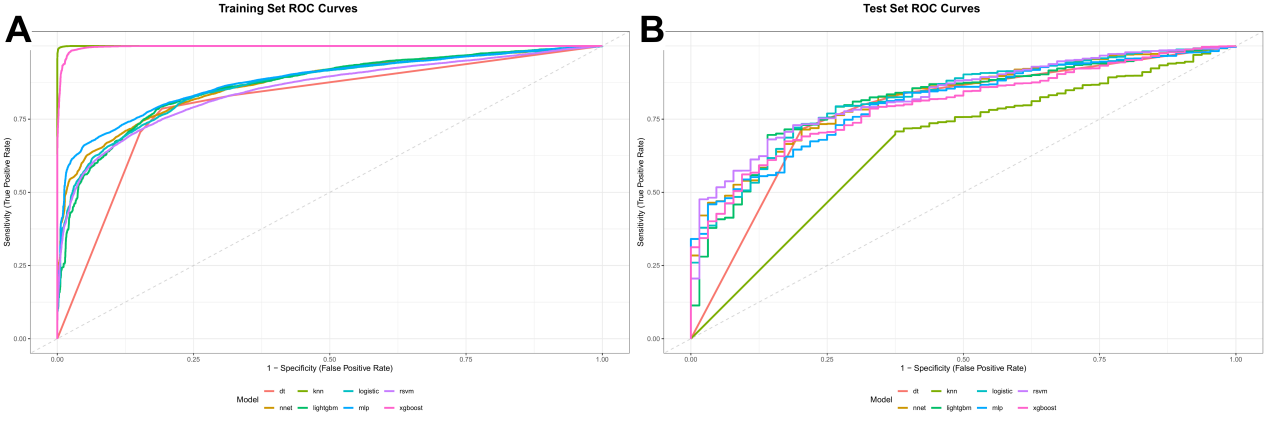
**

**Supplemental Figure S3. Receiver operating characteristic (ROC) curves showing the performance of the LightGBM (by five-fold cross-validation).**


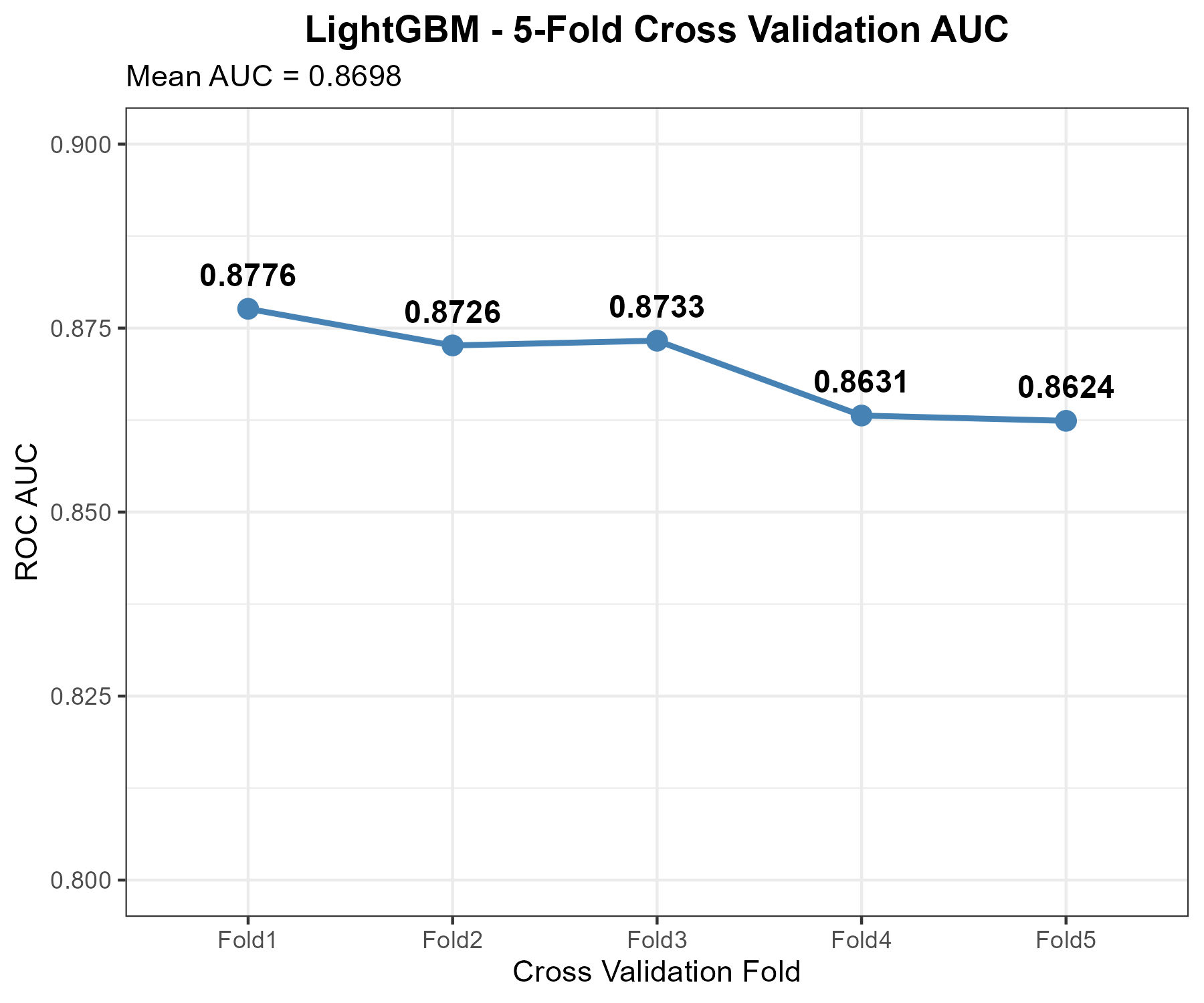


**Supplemental Figure S4. Performance comparison of our LightGBM model with other existing models.**

**
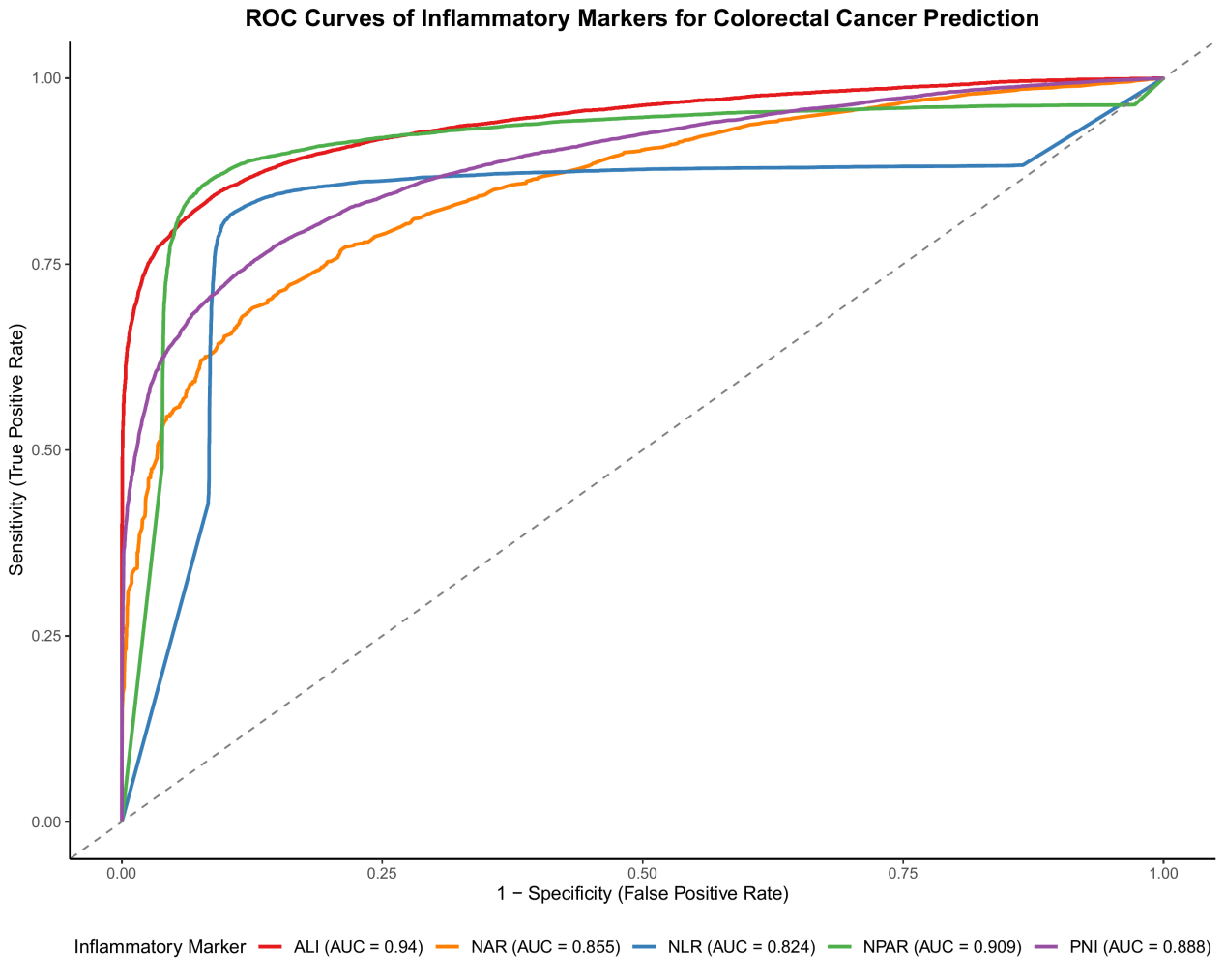
**

Note: ALI, Advanced lung cancer inflammation index.

NAR, Neutrophil-to-albumin ratio; neutrophil count (×10^9^)/ albumin (g/ L)

NLR,Neutrophil-to-lymphocyte ratio; absolute lymphocyte count (×10^9^)/absolute neutrophil count (×10^9^)

NPAR,Neutrophil percentage-to-albumin ratio; neutrophil percentage (%) × 100 / albumin (g/dL)

PNI,[Prognostic nutritional index; albumin (g/L) + 5×lymphocyte count (×10](https://pubmed.ncbi.nlm.nih.gov/38390093/)^[9](https://pubmed.ncbi.nlm.nih.gov/38390093/)^[)](https://pubmed.ncbi.nlm.nih.gov/38390093/)

:

**Supplemental Figure S5. Performance comparison of our LightGBM model in different subgroups.**


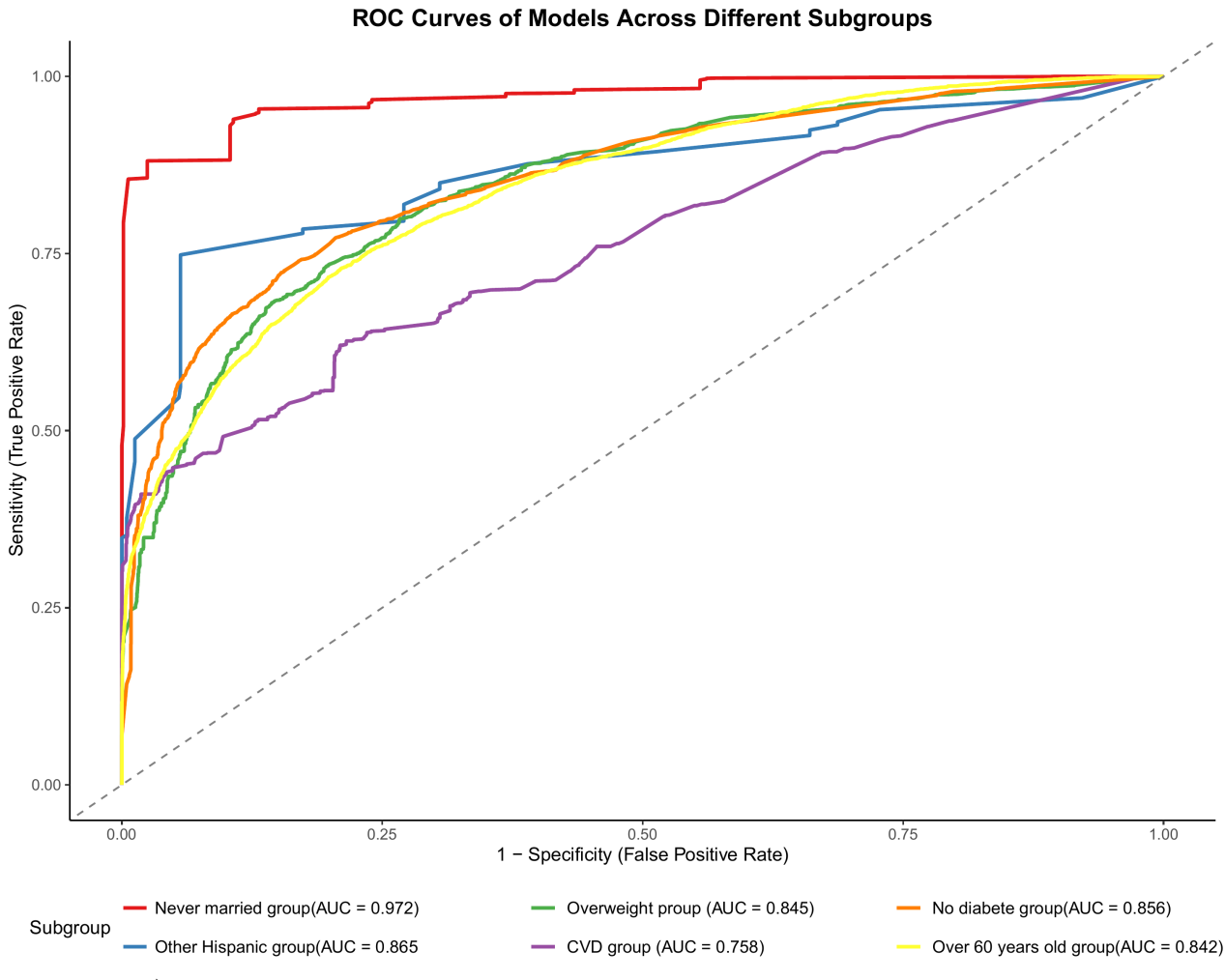


**Supplementary Table S1: Association between Log-ALI and colorectal cancer by including the NHANES with missing data not handling.**

| **ALI** |  | **Model I**  **[OR (95% CI)]** | ***p-value*** | **Model II**  **[OR (95% CI)]** | ***p-value*** | **Model III**  **[OR (95% CI)]** | ***p-value*** |
| --- | --- | --- | --- | --- | --- | --- | --- |
| **Per ln-unit increase** |  | 0.552(0.430,0.709) | **<.001** | 0.770(0.610,0.972) | **0.028** | 0.776(0.615,0.980) | **0.033** |
| **Q1** |  | Reference |  |  |  | Reference |  |
| **Q2** |  | 0.729(0.465,1.144) | 0.168 | 0.951(0.601,1.505) | 0.830 | 0.960(0.609,1.512) | 0.859 |
| **Q3** |  | 0.643(0.440,0.940) | **0.023** | 0.912(0.621,1.339) | 0.636 | 0.936(0.642,1.363) | 0.727 |
| **Q4** |  | 0.415(0.271,0.636) | **<.001** | 0.614(0.400,0.943) | **0.026** | 0.612(0.400,0.936) | **0.024** |

Note:

Abbreviation: ALI: advanced lung cancer inflammation index; NHANES, National Health and Nutrition Examination Survey; BMI, Body mass index; CVD, cardiovascular disease. OR, odds ratio; CI, confidence interval.

Model I was unadjusted.

Model II was adjusted for age, race, gender, poverty level, education and marital status.

Model III was adjusted for age, race, gender, poverty level, education, marital status, BMI, smoking status, alcohol consumption, hypertension, diabetes, and CVD.

**Supplementary Table S2: Association between Log-ALI and colorectal cancer by including the NHANES after imputation.**

| **ALI** |  | **Model I**  **[OR (95% CI)]** | ***p-value*** | **Model II**  **[OR (95% CI)]** | ***p-value*** | **Model III**  **[OR (95% CI)]** | ***p-value*** |
| --- | --- | --- | --- | --- | --- | --- | --- |
| **Per ln-unit increase** |  | 0.571(0.459,0.713) | **<.001** | 0.778(0.613,0.988) | **0.039** | 0.779(0.612,0.993) | **0.043** |
| **Q1** |  | Reference |  | Reference |  | Reference |  |
| **Q2** |  | 0.732(0.534,0.998) | **0.050** | 0.932(0.667,1.295) | 0.677 | 0.942(0.676,1.305) | 0.719 |
| **Q3** |  | 0.624(0.448,0.862) | **0.005** | 0.898(0.630,1.269) | 0.546 | 0.916(0.643,1.293) | 0.621 |
| **Q4** |  | 0.444(0.305,0.635) | **<.001** | 0.658(0.438,0.973) | **0.040** | 0.660(0.439,0.974) | **0.040** |

Note: ALI: advanced lung cancer inflammation index.

Weighted multivariable linear and logistic regression analyses were performed with ALI as the main independent variable and odds ratios (OR) were calculated to assess the association between ALI and colorectal cancer in Model I (unadjusted), Model II (adjusted for age, race, gender, poverty level, education and marital status), and Model III (fully adjusted for all covariates).

**Supplementary Table S3: Association between Log-ALI and colorectal cancer by excluding the lower 2.5% and upper 97.5% ALI value.**

| **ALI** |  | **Model I**  **[OR (95% CI)]** | ***p-value*** | **Model II**  **[OR (95% CI)]** | ***p-value*** | **Model III**  **[OR (95% CI)]** | ***p-value*** |
| --- | --- | --- | --- | --- | --- | --- | --- |
| **Per ln-unit increase** |  | 0.987(0.981,0.992) | **<.001** | 0.993(0.988,0.998) | **0.007** | 0.993(0.988,0.998) | **0.004** |
| **Q1** |  | Reference |  | Reference |  | Reference |  |
| **Q2** |  | 0.762(0.459,1.265) | 0.292 | 1.007(0.605,1.673) | 0.980 | 1.010(0.611,1.669) | 0.969 |
| **Q3** |  | 0.764(0.501,1.163) | 0.208 | 1.108(0.730,1.684) | 0.627 | 1.123(0.746,1.691) | 0.577 |
| **Q4** |  | 0.322(0.196,0.529) | **<.001** | 0.486(0.292,0.811) | **0.006** | 0.479(0.290,0.790) | **0.007** |

Supplementary Table S6. Performance metrics of our LightGBM model  in other existing models

| Biomarker | AUC | Accuracy | Sensitivity | Specificity |
| --- | --- | --- | --- | --- |
| ALI | 0.9398 | 0.876 | 0.8374 | 0.9156 |
| NPAR | 0.9089 | 0.8885 | 0.8562 | 0.9216 |
| PNI | 0.8875 | 0.8125 | 0.7692 | 0.8569 |
| NAR | 0.855 | 0.7792 | 0.7236 | 0.8361 |
| NLR | 0.8236 | 0.8552 | 0.8213 | 0.8899 |

Supplementary Table S7. Performance metrics of our LightGBM model in different subgroups.

| **Subgroup** | **AUC** | **Accuracy** | **Sensitivity** | **Specificity** |
| --- | --- | --- | --- | --- |
| Never married group | 0.972 | 0.794 | 0.985 | 0.865 |
| Other Hispanic group | 0.865 | 0.643 | 0.928 | 0.945 |
| No diabete group | 0.856 | 0.773 | 0.791 | 0.838 |
| Overweight proup | 0.845 | 0.761 | 0.768 | 0.865 |
| Over 60 years old group | 0.842 | 0.808 | 0.367 | 0.980 |
| CVD group | 0.758 | 0.762 | 0.453 | 0.940 |
